# l‐Citrulline Supplementation‐Increased Skeletal Muscle PGC‐1α Expression Is Associated with Exercise Performance and Increased Skeletal Muscle Weight

**DOI:** 10.1002/mnfr.201701043

**Published:** 2018-06-25

**Authors:** Myra O. Villareal, Toshiya Matsukawa, Hiroko Isoda

**Affiliations:** ^1^ Faculty of Life and Environmental Sciences University of Tsukuba Tsukuba City 305‐8572 Japan; ^2^ Alliance for Research on Mediterranean and North Africa University of Tsukuba Tsukuba City 305‐8572 Japan; ^3^ Graduate School of Life and Environmental Sciences University of Tsukuba Tsukuba City 305‐8572 Japan

**Keywords:** exercise performance, l‐citrulline, *PGC‐1α*, skeletal muscle weight, supplementation

## Abstract

**Scope:**

l‐citrulline has recently been reported as a more effective supplement for promoting intracellular nitric oxide (NO) production compared to l‐arginine. Here, the effect of l‐citrulline on skeletal muscle and its influence on exercise performance were investigated. The underlying mechanism of its effect, specifically on the expression of skeletal muscle peroxisome proliferator‐activated receptor‐gamma coactivator‐1α (PGC‐1α), was also elucidated.

**Methods and results:**

Six‐week‐old ICR mice were orally supplemented with l‐citrulline (250 mg kg^−1^) daily, and their performance in weight‐loaded swimming exercise every other day for 15 days, was evaluated. In addition, mice muscles were weighed and evaluated for the expression of PGC‐1α and PGC‐1α‐regulated genes. Mice orally supplemented with l‐citrulline had significantly higher gastrocnemius and biceps femoris muscle mass. Although not statistically significant, l‐citrulline prolonged the swimming time to exhaustion. PGC‐1α upregulation was associated with vascular endothelial growth factor α (*VEGFα*) and insulin‐like growth factor 1 (*IGF‐1*) upregulation. *VEGFα* and *IGF‐1* are important for angiogenesis and muscle growth, respectively, and are regulated by PGC‐1α. Treatment with NG‐nitro‐l‐arginine methyl ester hydrochloride (l‐NAME), a nitric oxide synthesis inhibitor, suppressed the l‐citrulline‐induced *PGC‐1α* upregulation in vitro.

**Conclusion:**

Supplementation with l‐citrulline upregulates skeletal muscle PGC‐1α levels resulting in higher skeletal muscle weight that improves time to exhaustion during exercise.

## 1 Introduction

Regular exercise and being active physically enhance endurance during exercise and more importantly, improve metabolic dysfunction that may help prevent lifestyle‐related diseases.^1^, ^2^ Physical inactivity increases the risk of development of obesity, type 2 diabetes, sarcopenia, hypertension, and cardiovascular diseases.^3^ Physically active people's life expectancy, without long‐standing illness, is in fact 8–10 years longer compared to that of inactive people.^4^ Several studies have demonstrated that enhancement of the skeletal muscle functions is observed to be the major positive impact of exercise.^1^, ^2^ This means that enhancing the skeletal muscle functions, in terms of improved mitochondrial biogenesis, capillaries, and fatty acid transporters, significantly improve exercise capacity.

Peroxisome proliferator‐activated receptor‐gamma coactivator‐1α (PGC‐1α) is the transcriptional coactivator responsible for the regulation of mitochondrial biogenesis, angiogenesis, oxidative metabolism, and muscle growth, and has a crucial role in the adaptation of muscles to exercise.^5^, ^6^ Muscle‐specific overexpression of PGC‐1α increases exercise capacity and improves maximal oxygen uptake (VO_2max_) by increasing mitochondrial biogenesis and capillary density in skeletal muscle.^7^ Skeletal muscle PGC‐1α is known to have an important role in exercise adaptation and enhancement of exercise capacity.^8^, ^9^

Nitric oxide (NO) is a reactive nitrogen molecule synthesized enzymatically through the catalytic action of nitric oxide synthase (NOS). NO is expressed in the skeletal muscles of mammals and acts as a second messenger in transduction pathways associated with the expression of genes for oxidative metabolism, vasodilation, and skeletal muscle contraction. NOS inhibition also reduces the maximal oxygen uptake during exercise in humans.^10^ Conversely, increased intracellular NO production leads to phosphorylation of CREB that in turn induces PGC‐1α expression.^11^ Thus, several studies have indicated that the physiological amount of NO positively impacts adaptation to exercise, much so that nitrate supplementation has been considered to be an ergogenic supplementation for athletes or in sports.^12^, ^13^

Normally, physiological NO is derived from l‐arginine. However, absorption of orally administered l‐arginine is hampered by first‐pass and systemic metabolism. l‐citrulline, on the other hand, is only subject to systemic metabolism. l‐citrulline, a nonessential amino acid, has recently been recognized as an effective alternative source of NO, and therefore can be taken as a dietary supplement to increase intracellular NO production.^14^ Oral supplementation with l‐citrulline increases NO levels by increasing endothelial NO synthase (eNOS) expression that results in improved endothelial function.^15^, ^16^ Neuronal NOS (nNOS) and eNOS are also expressed in the skeletal muscle.^11^ l‐citrulline is a precursor of l‐arginine but has several functional advantages over l‐arginine.^14^, ^15^ l‐citrulline is superior to l‐arginine in terms of ease of handling and palatability since it is tasteless, odorless, and non‐hygroscopic, whereas l‐arginine tends to be extremely bitter and highly water absorbent.^17^ Therefore, l‐citrulline is considered to be more effective in enhancing exercise performance by virtue of its effect on skeletal muscle regulation. Several studies have reported that l‐citrulline supplementation enhances exercise performance^18^, ^19^, ^20^; however, the underlying mechanism of its effect on skeletal muscles and exercise performance, has yet to be elucidated. In this study, the effect of l‐citrulline supplementation on exercise and the underlying cause of those effects were investigated in vivo, using exercised mice, and in vitro, using skeletal muscle cells.

## 2 Experimental Section

### 2.1 Chemicals

Rhodamine 123, l‐NAME, and sodium dodecyl sulfate (SDS) were purchased from Wako (Tokyo, Japan). ISOGEN was purchased from Nippongene (Tokyo, Japan). DMEM, RIPA buffer, protease inhibitor cocktail, and β‐Actin antibody were purchased from Sigma (MO, USA). Fetal bovine serum (FBS) and house serum (HS) were purchased from Gibco (NY, USA). MTT or 3‐(4,5‐dimethylthiazol‐2‐yl)‐2,5‐diphenyltetrazolium bromide and l‐NAME were purchased from Dojindo (Kumamoto, Japan). l‐citrulline was supplied by Kyowa Hakko Bio Co., Ltd., Tokyo, Japan) while PGC‐1α (3G6) antibody was purchased from Cell Signaling Technology (Hertfordshire, UK). Glyceraldehyde 3‐phosphate dehydrogenase (GAPDH) antibody (6C5) was purchased from Santa Cruz Biotechnology (CA, USA).

### 2.2 Animal Experiments

Five‐week‐old male ICR mice were obtained from Charles River Laboratories (Kanagawa, Japan) and maintained under a 12‐h light/dark cycle, with free access to water and a normal diet (MF, Oriental Yeast Co., Ltd., Japan). Body weight was measured daily while food intake was measured once a week. After 1 week of acclimatization, the mice were divided into three groups. Group 1 (“no exercise” group) was orally administered with distilled water (D.W.) without swimming exercise (*n* = 6). Group 2 (control group) was orally administered with water. Group 3 (l‐citrulline group) was orally administered with 250 mg kg^−1^ day of l‐citrulline. The l‐citrulline dose used in this study was based on Takeda et al.’s^21^ study. Both Groups 2 and 3 performed swimming exercises (*n* = 7 per group). Oral administration of 100 μL sample solution was done using animal‐feeding needles (Group 1 and 2: D.W. or Group 3: 250 mg kg^−1^ l‐citrulline dissolved in water) every day for 15 days, 1 h before the swimming exercise, and at the same time on “no exercise” days. The exercise protocol was adapted from Takeda et al.^21^ with some modifications. Briefly, mice with a load corresponding to 5% of their body weight attached to their tails, were trained to perform the swimming exercise for 10 min, in a tank (30 × 30 × 40 cm) filled with water to a depth of 25 cm., with water temperature kept at 30 ± 1 °C. The swimming exercise was performed every other day for 14 days. On day 15, the mice were made to swim to exhaustion with a load corresponding to 10% of their body weight. Each mouse was considered to have reached its point of exhaustion when it failed to raise its face from the water surface to breathe within a period of 5 s. Blood lactate levels were measured before and after exercise (0 and 60 min) using Lactate Pro 2 (Arkrey, Japan). Blood glucose levels were measured after exercise (0 min) using Glucose Pilot system (Iwai Chemicals Company, Japan). The mice were sacrificed and blood and tissues from the liver, gastrocnemius, and biceps femoris were collected. The serum was separated from the blood by centrifugation at 3000 × *g* for 10 min and the serum biochemical parameters (BUN, creatinine, total ketone bodies, AST, ALT, ALP, nonessential fatty acid [NEFA]) were analyzed by Oriental Yeast Co., Ltd., (Japan) using test kits obtained from Wako Pure Chemical Industries (Osaka, Japan). All animal experiments performed are in compliance with the guidelines and regulations for Animal Experiments of the University of Tsukuba (No. 16‐044), and were approved by the International Animal Care and Use Committee of the University of Tsukuba.

### 2.3 Real‐Time PCR Analysis

Total RNA was isolated from tissue samples (50 mg) and C2C12 myotubes using ISOGEN. For C2C12 myotubes, cells were treated with or without l‐citrulline or NOS inhibitor l‐NAME. Total RNA isolation and TaqMan real‐time PCR amplification reactions were performed as previously reported.^22^ For the quantification of the gene expression in muscle tissues and C2C12 myotubes, the following specific TaqMan probes purchased from Applied Biosystems (CA, USA) were used: *β‐actin* (Mm00607939_s1), *PGC‐1α* (Mm01208835_m1), *LDHa* (Mm01612132_g1), *LDHb* (Mm01267402_m1), *MCT1* (Mm01306379_m1), *CPT‐1β* (Mm00487191_g1), *TFAM* (Mm00447485_m1), vascular endothelial growth factor α (*VEGFα*) (Mm00437306_m1), and insulin‐like growth factor 1 (*IGF‐1*) (Mm00439560_m1). The mRNA levels of all genes were normalized to *β‐actin* mRNA levels (internal control).

### 2.4 Western Blotting

Total protein was isolated from tissue samples (10 mg) using RIPA buffer containing a protease inhibitor cocktail according to the manufacturer's instructions. Protein samples (15 μg) were separated using 10% SDS‐PAGE and transferred to a PVDF membrane (Merck Millipore, USA). Membranes were incubated with primary antibody at 4 °C overnight, then washed, and incubated with secondary antibodies (IRDye 800CW donkey antirabbit IgG or IRDye 680LT goat antimouse [LI‐COR, Inc., NE, USA]) at room temperature for 30 min. The signal was detected using the Odyssey Fc Imaging System (LI‐COR, Inc., NE, USA).

### 2.5 Cell Culture and Differentiation

The mouse C2C12 myoblasts (ATCC, USA) were cultured in DMEM supplemented with 10% FBS and 1% penicillin (5000 μg mL^−1^)–streptomycin (5000 IU mL^−1^) (Lonza, Tokyo, Japan). To induce C2C12 myoblasts to differentiate into C2C12 myotubes, C2C12 cells were cultured until confluent and then transferred to DMEM containing 2% horse serum, and incubated further for 5 days with the growth medium changed every other day.

### 2.6 MTT Assay

Following treatment and incubation with l‐citrulline at different concentrations, MTT solution (5 mg mL^−1^) was added to the C2C12 myotubes culture and incubated further for 3 h until formazan crystals were formed. Formazan crystals were then dissolved by adding 10% SDS and the plates incubated further for 16 h. The absorbance at 570 nm was measured using a Powerscan HT plate reader (Dainippon Sumitomo Pharma Co, Ltd., Japan).

### 2.7 Statistical Analysis

All the results are expressed as the mean ± standard deviation, and statistical evaluation was performed using the Student's *t*‐test when two value sets were compared. Analysis that includes multiple comparisons were carried out using one‐way analysis of variance or ANOVA, followed by Tukey's multiple comparison test using SPSS (IBM Statistics for Windows, version 22.0. IBM Corp., Armonk, NY). *p* ≤ 0.05 was considered to be statistically significant.

## 3 Results

### 3.1 l‐Citrulline Supplementation before Exercise Prevented Exercise‐Induced Elevation of Blood Lactate and Decrease in Glucose Levels

**Figure 1:**
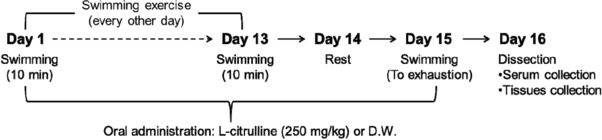
Study design. Mice were made to perform a swimming exercise every other day for 14 days. A swimming‐until‐exhaustion test was carried out on day 15. During the experimental period, mice were orally administrated with l‐citrulline (250 mg kg^−1^) or distilled water (D.W.) every day.

**Figure 2:**
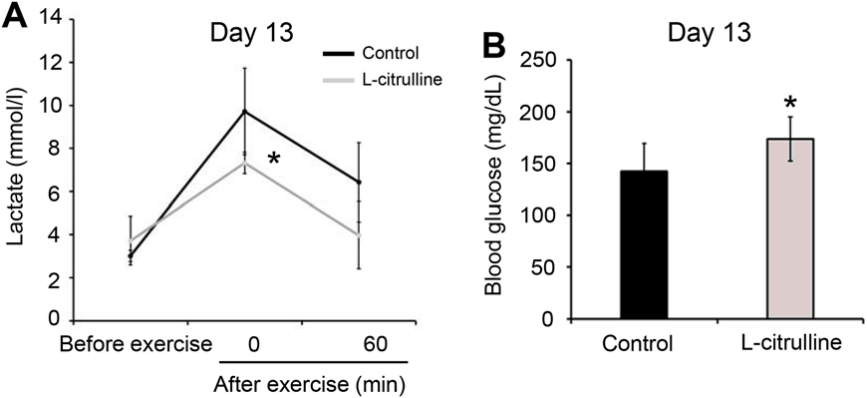
Effect of l‐citrulline supplementation on the blood lactate and glucose levels after weight‐loaded exercise performance. Mice orally administrated with l‐citrulline (250 mg kg^−1^) or distilled water (D.W.) were made to perform a weight‐loaded forced swimming test for 10 min on day 13. A) Blood lactate levels before and after exercise (0 and 60 min) and B) blood glucose levels after exercise (0 min) were evaluated. Values are expressed as the mean ± standard deviation. *p ≤ 0.05 and **p ≤ 0.01 indicate a significant difference compared to the control group.

To examine the effect of l‐citrulline supplementation on blood lactate and glucose levels during exercise, mice were orally administered with l‐citrulline or water (control), 1 h before exercise, for 15 days. On day 13, mice were made to perform weight‐loaded forced swimming test for 10 min, and 1 h later, were given l‐citrulline supplementation (**Figure** 1). Changes in the blood lactate levels during exercise are associated with exercise performance. Lactate that accumulates in the muscles produces H^+^ ions that cause fatigue, impairing muscle function and performance.^23^ As shown in **Figure** 2A, before exercise, the blood lactate levels between the control and l‐citrulline groups were not significantly different. However, after exercise, the lactate level of the l‐citrulline group was lowered (9.7 ± 2.0 mM vs. 7.3 ± 0.5 mM, *p* = 0.055). The difference in the lactate levels before and after exercise however, was bigger in the control group. Lactate is produced when glucose or glycogen is used as a fuel source, so that an increase in the glucose levels would lead to higher lactate levels. Additionally, the l‐citrulline group had significantly higher glucose levels after exercise on day 13 compared to the control (142 ± 26.8 mg dL^−1^ vs. 174 ± 21.5 mg dL^−1^, *p* = 0.04) (Figure 2B).

### 3.2 l‐Citrulline Supplementation before Exercise Increased Muscle Weight and Increased Time to Exhaustion in Swimming

**Figure 3:**
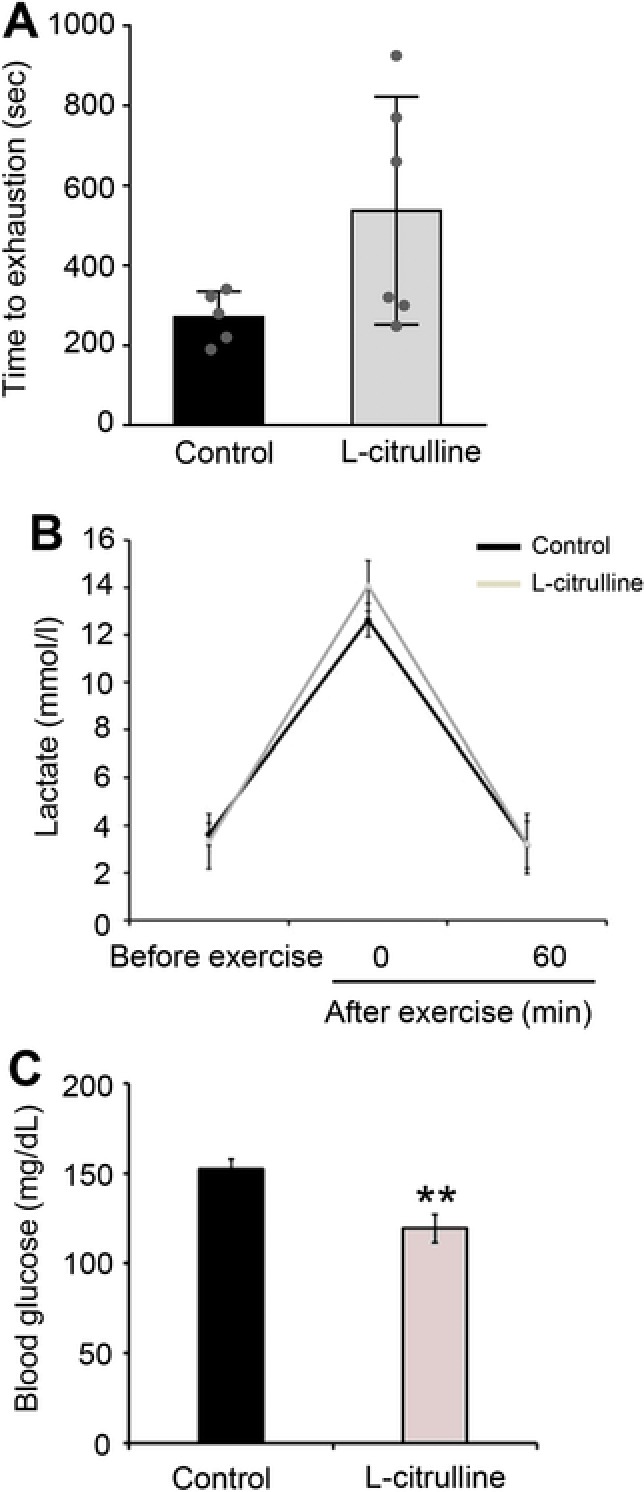
Effect of l‐citrulline supplementation on swimming endurance. Mice were trained to perform swimming exercise every other day for 14 days, then a swimming‐until‐exhaustion test was carried out on day 15. A) Swimming time to exhaustion, B) blood lactate levels before and after exercise (0 and 60 min), and C) blood glucose levels after exercise (0 min) were evaluated. Values are expressed as the mean ± standard deviation. *p ≤ 0.05 and **p ≤ 0.01 indicate a significant difference compared to the control group. The individual raw data was plotted over the bar graph.

To evaluate the effect of l‐citrulline supplementation on exercise performance, mice were made to perform a weight‐loaded forced swimming test until exhaustion, with a load corresponding to 10% of their body weight, 1 h after l‐citrulline supplementation (Figure 1). As shown in **Figure** 3A, the l‐citrulline group had longer time to exhaustion, though it was not statistically significant, compared to that of the control groups (270 ± 64.4 s vs. 537 ± 285.2 s, *p* = 0.07). Although the mice in the l‐citrulline supplementation group swam for a longer period of time than the control group, the lactate levels between these groups were not significantly different (12.6 ± 0.7 mM vs. 14.0 ± 2.6 mM, *p* = 0.27) (Figure 3B). Also, compared to the control, the l‐citrulline group had significantly lower blood glucose levels (153 ± 5.4 mg dL^−1^ vs. 119 ± 7.9 mg dL^−1^, *p* < 0.01) (Figure 3C). Additionally, l‐citrulline supplementation showed an increase in the weight of the gastrocnemius (0.23 ± 0.02 g vs. 0.28 ± 0.02 g, *p* = 0.02) and biceps femoris muscles (0.34 ± 0.01 g vs. 0.52 ± 0.07 g, *p* < 0.01) compared to the control, even though the body weights of both groups were not significantly different (**Table** 1). The food intake, including the amino acids from feeds, was also not significantly different between the control and the l‐citrulline‐administered groups (data not shown). Although other studies have reported that l‐citrulline elevates the levels of urea nitrogen, creatinine, and total ketone bodies in the blood after exercise,^24^, ^25^ this study shows that l‐citrulline supplementation lowered the creatinine level (0.20 ± 0.05 mg dL^−1^ vs. 0.14 ± 0.02 mg dL^−1^, *p* = 0.06) and total ketone bodies (1163.3 ± 245.7 μM vs. 948.4 ± 206.6 μM, *p* = 0.15) over time (**Table** 2).

**Table 1:** Mice body weight, food intake, and tissue weight

		Swimming	
	No exercise	Control	l‐citrulline
Initial body weight [g]	28.7 ± 1.3	28.2 ± 1.4	28.5 ± 0.8
Final body weight [g]	30.7 ± 1.2	31.6 ± 2.0	32.5 ± 1.7
Food intake [g day^−1^]	3.67 ± 0.12	3.64 ± 0.23	3.92 ± 0.32#
Kidney [g]	0.43 ± 0.01	0.50 ± 0.03**	0.45 ± 0.02#
Liver [g]	1.19 ± 0.04	1.18 ± 0.04	1.21 ± 0.06
Gastrocnemius [g]	0.24 ± 0.03	0.23 ± 0.02	0.28 ± 0.02* ^,^ ##
Biceps femoris [g]	0.26 ± 0.03	0.34 ± 0.01**	0.52 ± 0.07** ^,^ #

Values are expressed as the mean ± standard deviation. ^*^ *p* ≤ 0.05 and ^**^ *p* ≤ 0.01 indicate a significant difference from the “no exercise” group. ^#^ *p* ≤ 0.05 and ^##^ *p* ≤ 0.01 indicate a significant difference compared to the control group.

**Table 2:** Level of serum biochemical parameters at the end of the study

		Swimming	
	No exercise	Control	l‐citrulline
BUN [mg dL^−1^]	32.3 ± 0.9	23.9 ± 2.1**	23.3 ± 2.7**
Creatinine [mg dL^−1^]	0.15 ± 0.01	0.20 ± 0.05	0.14 ± 0.02
Total ketone bodies [μM]	593.3 ± 53.3	1163.3 ± 245.7**	948.4 ± 206.6*
AST [IU L^−1^]	1782.5 ± 280.9	1922.0 ± 461.2	1750.4 ± 238.1
ALT [IU L^−1^]	226.3 ± 3.5	226.8 ± 40.4	201.7 ± 23.7
ALP [IU L^−1^]	345.0 ± 17.9	433.7 ± 37.6**	369.4 ± 40.9#
NEFA [μEq L^−1^]	1125.8 ± 140.9	1298.2 ± 129.8	1180.3 ± 158.1

Values are expressed as the mean ± standard deviation. ^*^ *p* ≤ 0.05 and ^**^ *p* ≤ 0.01 indicate a significant difference compared to the “no exercise” group. ^#^ *p* ≤ 0.05 indicates a significant difference compared to the control group. BUN, urea nitrogen; AST, aspartate transaminase; ALT, alanine transaminase; ALP, alkaline phosphatase; NEFA, non‐esterified fatty acid.

### 3.3 l‐Citrulline Supplementation Upregulated the PGC‐1α Expression in Gastrocnemius and Biceps Femoris

**Figure 4:**
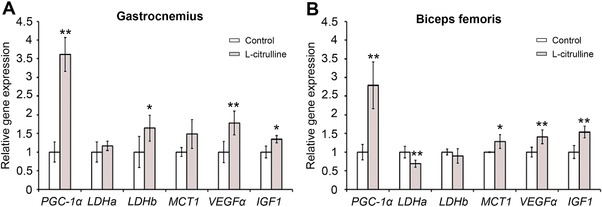
Effect of l‐citrulline supplementation on the expression of *PGC‐1α* and PGC‐1α‐related genes in the gastrocnemius and biceps femoris. Expression levels of *PGC‐1α* and PGC‐1α‐targeted genes in the A) gastrocnemius and B) biceps femoris were evaluated. Expression levels of mRNA were normalized to the *β‐actin* expression level. Values are expressed as the mean ± standard deviation and relative to the “no exercise” group. *p ≤ 0.05 and **p ≤ 0.01 indicate a significant difference compared to the control group.

**Figure 5:**
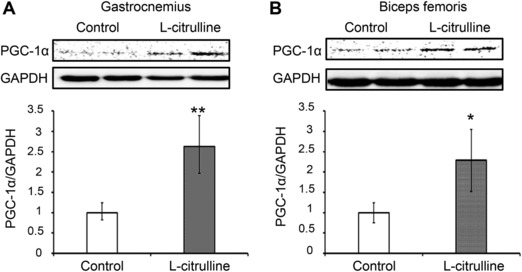
Effect of l‐citrulline supplementation on the protein expression of PGC‐1α in the gastrocnemius and biceps femoris. Protein expression levels of PGC‐1α in the A) gastrocnemius and B) biceps femoris were evaluated. All gels were run under the same experimental conditions and the representative blots were shown. The protein expression levels were normalized to GAPDH expression. Values are expressed as the mean ± standard deviation and relative to the unexercised group. *p ≤ 0.05 and **p ≤ 0.01 indicate a significant difference from the control group.

PGC‐1α in the skeletal muscle has significant regulatory role in the muscles’ several adaptations to exercise such as lactate metabolism, angiogenesis, and muscle growth.^1^, ^5^ Since the gastrocnemius and biceps femoris are extensively used during swimming, we specifically evaluated the effects of l‐citrulline supplementation on the gastrocnemius and biceps femoris. Supplementation with l‐citrulline greatly increased *PGC‐1α* expression in the gastrocnemius (**Figure** 4A; 3.6 ± 0.5‐fold, *p* < 0.01) and biceps femoris (Figure 4B; 2.8 ± 0.6‐fold, *p* < 0.01). As shown in **Figure** 5, the protein expression level of PGC‐1α in the gastrocnemius and biceps femoris (2.6 ± 0.7‐fold, *p* < 0.01 and 2.3 ± 0.8‐fold, *p* = 0.02, respectively) was also increased with l‐citrulline supplementation. VEGFα and *IGF‐1*, cytokines released from skeletal muscle are important factors for angiogenesis and muscle growth, respectively, and are under the regulation of PGC‐1α.^5^ As shown in Figure 4, l‐citrulline‐supplemented mice had significantly higher levels of *VEGFα* and *IGF‐1* in their gastrocnemius (1.8 ± 0.3‐fold, *p* < 0.01 and 1.3 ± 0.1‐fold, *p* < 0.01, respectively) and biceps femoris (1.4 ± 0.2‐fold, *p* < 0.01 and 1.5 ± 0.2‐fold, *p* < 0.01, respectively) compared to the control group. The role of PGC‐1α in the promotion of lactate metabolism through increased lactate dehydrogenase (LDH) B and monocarboxylate transporter 1 (MCT1) expression in the skeletal muscle has already been established.^26^ l‐citrulline supplementation also increased the *MCT1* expression in the gastrocnemius (1.5 ± 0.4‐fold, *p* = 0.05) and biceps femoris (1.3 ± 0.2‐fold, *p* < 0.05). The upregulation of *LDH B*, however, was only observed in the gastrocnemius (1.6 ± 0.3‐fold, *p* < 0.01) (Figure 4).

### 3.4 l‐Citrulline‐Induced *PGC‐1α* Upregulation in C2C12 Myotubes was Suppressed by NO Synthesis Inhibition

**Figure 6:**
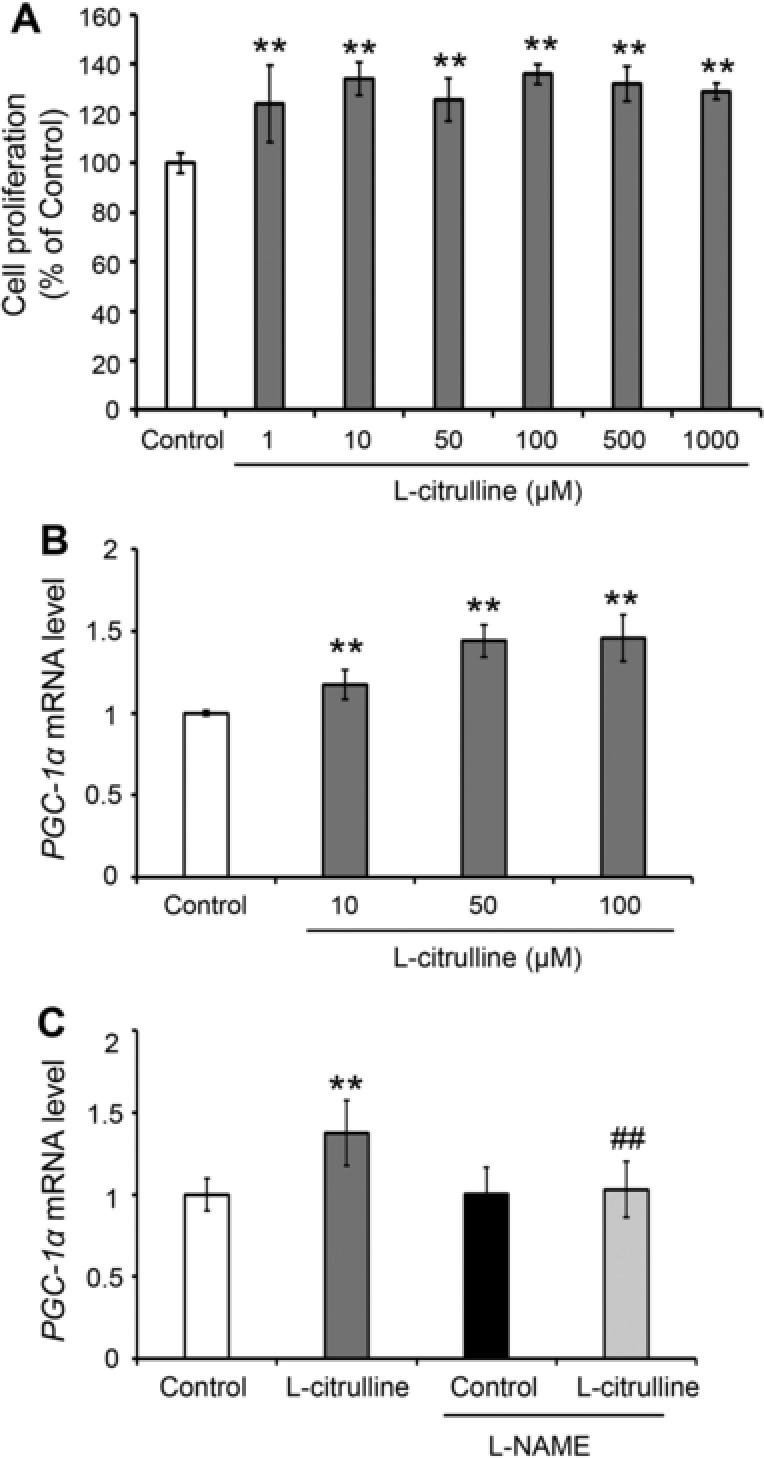
Effect of l‐citrulline on the gene expression of *PGC‐1α* in C2C12 myotubes. Differentiated C2C12 myotubes were treated with or without l‐citrulline for 24 h. A) After that, cell viability was evaluated and value expressed as percentage (%) of control. B) C2C12 myotubes were treated with or without l‐citrulline (10, 50, 100 μM) for 1 h. C) l‐citrulline (50 μM) treatment was performed with or without 100 μM NG‐nitro‐l‐arginine methyl ester hydrochloride (l‐NAME) for 1 h. Following treatment, *PGC‐1α* mRNA levels were quantified using real‐time PCR and the values normalized to the expression level of *β‐actin*. Values are expressed as the mean ± standard deviation of triplicate experiments. **p ≤ 0.01 indicates a significant difference from the control group. ^##^ *p* ≤ 0.01 indicate a significant difference from the l‐NAME‐treated l‐citrulline group.

To investigate the effect of l‐citrulline on the expression of *PGC‐1α* in the skeletal muscle cells, the effective and non‐cytotoxic concentrations of citrulline were first determined by performing MTT assay using C2C12 myotubes, a cellular model of skeletal muscle. l‐citrulline treatment significantly increased the proliferation of C2C12 myotubes but a gradual decrease was observed starting at concentrations of more than 500 μM l‐citrulline (**Figure** 6A). Considering the MTT assay results, 100 μM l‐citrulline was chosen as the best concentration to use in the succeeding experiments. Treatment with 10, 50, and 100 μM l‐citrulline for 1 h increased the *PGC‐1α* expression in C2C12 myotubes by 1.2 ± 0.1‐fold, 1.4 ± 0.1‐fold, and 1.5 ± 0.1‐fold, respectively (*p* < 0.01) (Figure 6B). l‐citrulline is known to elevate intracellular NO levels by promoting NOS activity.^14^, ^15^, ^16^ Consequently, an elevation of intracellular NO production increases muscle cell's *PGC‐1α* expression.^27^ In the current study, the upregulation of *PGC‐1α* expression in C2C12 myotubes by l‐citrulline treatment was suppressed in the presence of NOS inhibitor l‐NAME (Figure 6C).

## 4 Discussion

In the field of sports physiology, nitrate supplementation is believed to be an effective strategy for the enhancement of exercise performance. Increased intracellular NO production increases PGC‐1α expression via phosphorylation of CREB.^11^ The transcriptional activator PGC‐1α has an important role in the regulation of genes associated with adaptation to exercise such as oxidative metabolism, angiogenesis, and muscle growth.^1^ It has been reported that non‐protein amino acids, (e.g., ornithine, citrulline, and homocysteine) which are not used for protein synthesis, play an important role in cell metabolism.^28^

l‐citrulline, a non‐essential amino acid, has recently been demonstrated to be effective in increasing of intracellular NO production by increasing NOS expression.^14^, ^15^, ^16^ The major findings of this study are that the upregulation of PGC‐1α in the skeletal muscle by l‐citrulline is associated with increased exercise performance and muscles weight. l‐citrulline is a precursor of l‐arginine that is considered to be preferable than l‐arginine in terms of solubility and taste, as well as several functional advantages.^14^, ^15^, ^17^ Moreover, l‐arginine can enhance NO production but it is rapidly metabolized in the small intestine and liver when administered orally.^14^ Therefore, when compared to l‐citrulline, l‐citrulline comes out as a more effective sports supplement for enhancing sports performance compared to l‐arginine. Some studies, have reported that single supplementation of l‐citrulline does not help improve exercise performance^21^, ^29^ suggesting the need for long‐term supplementation of l‐citrulline (more than 1 week) to effectively enhance exercise tolerance.^20^, ^21^ The current study also provides data to validate assumptions of past researches that l‐citrulline supplementation for 15 days can enhance exercise performance and increase muscle mass. The results of this study therefore suggest that long‐term intake of l‐citrulline has a positive impact on exercise performance.

Lactate is produced during intense exercise when the supply of O_2_ is insufficient. The blood lactate level during exercise is dependent on the ratio of lactate production to lactate clearance.^23^, ^30^ Hydrogen ions (H^+^) dissociate from lactic acid and accumulate in the muscles, causing fatigue and depressing muscle function and contraction.^23^ In addition, decreased blood glucose levels (hypoglycemia) during exercise also causes fatigue and low energy, leading to exercise cessation.^31^ The l‐citrulline supplemented mice group had lower blood lactate levels and higher glucose levels immediately after exercising with the same load (Figure 2). These results therefore suggest that the observed inhibition of lactate production and/or increased lactate metabolism during exercise can be attributed to l‐citrulline supplementation. However, the blood lactate levels after exercise were not different from the after the swimming‐to‐exhaustion test blood lactate levels (Figure 3). Following a swimming‐to‐exhaustion test, the blood lactate levels gradually increase depending on the duration of the exercise.^32^ In this study, l‐citrulline supplemented group had longer average swimming to exhaustion time compared to the control (270 s vs. 537 s, respectively) (Figure 3A). Therefore, it can be assumed that blood lactate levels before and after the swimming‐to‐exhaustion test remained the same. Skeletal muscle PGC‐1α promotes lactate metabolism by increasing the expression of LDH B and MCT1, and conversely, prevents lactate production by suppressing the expression of LDH A that catalyzes the conversion of pyruvate to lactate.^1^, ^26^ A decrease in *LDH A* expression and increase in *MCT1* and *LDH B* levels were also observed following l‐citrulline supplementation (Figure 3). Several studies reported have established that l‐citrulline has antidiabetic and antiobesity effects^33^, ^34^, ^35^ but does not affect the blood glucose level. So, in this study, it can then be assumed that l‐citrulline did not affect the blood glucose levels and instead suggests that a decrease in lactate production and an increase in lactate metabolism are therefore involved in the regulation of blood lactate and glucose levels. PGC‐1α mRNA level is elevated after performing exercise but the level returns back to its “before exercise level” during the rest period, specifically within 24 h after exercise.^36^, ^37^ Even though the muscle samples were collected 24 h after exercise, an increase in PGC‐1α mRNA and protein expression due to l‐citrulline supplementation was still observed (Figure 2). Therefore, it is clear that the increase in lactate metabolism can be attributed to the longitudinal PGC‐1 α upregulation by l‐citrulline rather than a transient upregulation by exercise.

Increased blood flow enhances not only exercise performance, by improving nutrient and oxygen delivery in muscle, but also by boosting protein synthesis and muscle fiber repair.^38^ Several reports have suggested that l‐citrulline supplementation can improve blood pressure, VO_2_ kinetics, and exercise performance in healthy adults,^19^, ^20^ and believed to be due to PGC‐1α upregulation in the muscles that promoted formation of new blood vessels (angiogenesis), and thus, integrating oxygen/nutrient consumption and supply. PGC‐1α expression in cultured muscle cells and in skeletal muscle promotes the expression of several angiogenic factors, including VEGF, which plays a crucial role in vascular development. At the same time, muscle vascularization by VEGFα increases blood supply and oxygen availability in the muscle, increasing exercise time and endurance.^39^ In the current study, an l‐citrulline‐induced increase in skeletal muscle *VEGFα* expression was observed (Figure 4), suggesting that l‐citrulline supplementation‐induced angiogenesis in skeletal muscle can be associated with the observed increase in swimming time. It is a well‐known fact that oral supplementation with l‐citrulline elevates NO levels by increasing NOS expression, resulting in improved endothelial function.^15^ On the other hand, NOS inhibition has been established to reduce maximal oxygen uptake during exercise in humans.^13^ However, increased intracellular NO production induces PGC‐1α expression.^11^ In this study, l‐citrulline‐induced *PGC‐1α* upregulation in C2C12 myotubes was suppressed by l‐NAME, a NOS inhibitor (Figure 6), suggesting, therefore, that l‐citrulline‐increased skeletal muscle PGC‐1α level was due to the rise in the intracellular NO production.

Several PGC‐1α variants are expressed from alternative gene promoter, namely PGC‐1α‐b and PGC‐1α4, and have been shown to induce VEGF expression in skeletal muscle and angiogenesis.^40^ Transgenic expression of PGC‐1α4 in skeletal muscle in mice induces angiogenesis in vivo.^40^ PGC‐1α4 also activates a hypertrophic gene program in skeletal muscle. In addition, PGC‐1α4 also regulates skeletal muscle growth by inducing the anabolic hormone IGF‐1 and repressing myostatin, a powerful inhibitor of muscle differentiation and growth.^7^, ^41^ In this study, skeletal muscle weight was increased corresponding to PGC‐1α and IGF‐1 upregulation by l‐citrulline. It can therefore be inferred from these results that l‐citrulline induces PGC‐1α4 in association with l‐citrulline‐induced *VEGFα* and *IGF‐1* upregulation. Elevated PGC‐1α in muscle dramatically protects against the sarcopenia, obesity, and diabetes that normally accompanies aging.^42^ Therefore, the use of l‐citrulline to increase PGC‐1α expression may be useful in the management of diseases such as obesity, diabetes, and sarcopenia, as well as in the enhancement of exercise performance.

## 5 Concluding Remarks

l‐citrulline supplementation before exercise upregulates PGC‐1α expression in the skeletal muscle, resulting in a significant increase in skeletal muscle weight. A longer average time before mice became exhausted during exercise was also observed in l‐citrulline‐supplemented animals, but the significance of this effect needs further verification in a clinical trial. Furthermore, inhibition of NOS expression suppresses the l‐citrulline‐induced *PGC‐1α* upregulation. Further experiments would aim to compare the effect of other amino acids to the effect of l‐citrulline on enhancing exercise performance and increasing muscle weight. This study has demonstrated that l‐citrulline supplementation resulted in a significant improvement in exercise performance and increased skeletal muscle weight, and therefore may be used to enhance sports or exercise performance.

## Conflict of Interest

The authors declare no conflict of interest.
